# Supporting Visual Working Memory in Healthy Aging: A Review of Current Interventions and Perspectives on Future Approaches

**DOI:** 10.3390/brainsci16090981

**Published:** 2026-09-16

**Authors:** Virginia Tronelli, Alessandra Barbon, Veronica Mazza

**Affiliations:** Center for Mind/Brain Sciences (CIMeC), University of Trento, 38068 Rovereto, TN, Italy

**Keywords:** visual working memory, cognitive aging, cognitive training, non-invasive brain stimulation, game-based cognitive training, combined interventions, placebo and nocebo effects, expectation

## Abstract

**Highlights:**

**What are the main findings?**

- Current interventions suggest that visual working memory retains a degree of plasticity in healthy aging. Despite encouraging findings, intervention outcomes remain heterogeneous due to differences in intervention protocols, measures, and individual variability.
- Current evidence suggests that placebo and nocebo effects may influence cognitive performance. Although findings are promising, further research is needed to establish their efficacy and clarify the mechanisms underlying these effects in older adults.

**What are the implications of the main findings?**

- Placebo and nocebo effects may complement existing interventions targeting visual working memory in aging.
- Clarifying how psychosocial and contextual factors shape intervention outcomes could help optimize existing interventions and determine whether placebo/nocebo manipulations can ultimately improve their accessibility and resource efficiency.

**Abstract:**

Visual working memory (vWM) undergoes age-related decline, affecting storage capacity, representational precision, feature binding, and inhibitory control, with consequences for everyday functioning. Nevertheless, accumulating evidence indicates that the aging brain retains substantial plasticity, providing an opportunity of intervention. This review synthesizes current evidence on approaches to improving vWM in healthy older adults, including classic and game-based cognitive training, non-invasive brain stimulation (NIBS), and multimodal protocols that combine cognitive training with physical exercise or brain stimulation. Across the literature, process-based cognitive training produces improvements on trained and closely related tasks, whereas evidence for durable far-transfer remains limited. NIBS demonstrates promising neurophysiological effects, particularly when targeting frontoparietal networks, although behavioral outcomes remain heterogeneous and appear to depend on stimulation parameters and individual characteristics. Emerging findings further suggest that combining interventions may enhance selected aspects of vWM, highlighting the importance of personalized approaches. Despite these advances, intervention effects are often modest, task-specific, and resource-intensive. As a complementary approach, the review proposes placebo and nocebo effects as a theoretically grounded hypothesis that warrants empirical investigation for potentially complementing existing interventions and promoting healthy cognitive aging rather than confounds.

## 1. Introduction

Cognitive aging refers to the gradual and heterogeneous changes in mental abilities that occur across the lifespan as part of the natural process of growing older [1]. These changes can affect a wide range of cognitive functions and—even when subtle—may have meaningful consequences for everyday functioning, including health, independence, and social participation, ultimately shaping how successfully a person ages [1]. Importantly, cognitive aging does not entail a uniform decline across all domains: while some abilities, such as vocabulary knowledge and emotion regulation, remain relatively stable or may even improve with age, others show progressive deterioration [1,2].

Among the cognitive functions that decline with aging, visual working memory (vWM) appears particularly vulnerable, reflecting broader age-related changes in the encoding, maintenance, and control of visual representations. Older adults typically exhibit reduced vWM capacity, maintaining fewer visual objects simultaneously, especially under increasing memory load [3]. Aging also affects the quality of stored information, which becomes less detailed and precise, and more variable, leading to noisier and less reliable recall [3]. One explanation is that age-related declines in early perceptual processing—including reduced perceptual acuity and slower visual processing speed—compromise the encoding of visual information, resulting in memory representations that are already less accurate before maintenance begins [4,5,6,7]. At the same time, resource-based models propose that vWM relies on a limited pool of cognitive resources that must be shared across all stored representations: the more items need to be maintained in memory, the fewer resources can be allocated to each individual item, reducing representational precision [3]. In addition, older adults show impairments in feature binding, making associations between an object’s properties (e.g., color and location) more susceptible to confusion or misassignment, thereby reducing the stability of integrated visual representations [3,8]. Crucially, these representational deficits appear to be linked to age-related reductions in the ability to suppress irrelevant information. According to the inhibitory deficit hypothesis, aging weakens the ability to suppress irrelevant information, allowing distracting stimuli to enter WM and compete with goal-relevant representations [9]. Consistently, evidence indicates that older adults exhibit reduced inhibition of goal-irrelevant sensory information, making it more difficult to filter distractors and protect stored representations from interference or overwriting by incoming input [10].

Overall, these findings suggest that age-related decline in vWM cannot be attributed to a single underlying deficit, but rather reflects the combined disruption of perceptual, attentional, storage and inhibitory processes, resulting in visual representations that are fewer, less precise, weakly bound, and less effectively protected from interference. Given the central role of these mechanisms in a large proportion of everyday activities—such as locating objects in cluttered environments or remembering visual information—age-related alterations in vWM may have widespread consequences for functional independence and quality of life [11]. For this reason, research in cognitive neuroscience has devoted considerable effort to developing interventions to improve vWM.

## 2. Current Approaches for Supporting Visual Working Memory in Aging

Despite these age-related changes, a substantial body of behavioral and neuroscientific research suggests that older adults retain a considerable capacity for adaptation. Rather than reflecting a purely passive decline, cognitive aging is increasingly viewed as a dynamic process in which compensatory mechanisms help mitigate the functional impact of age-related neural and cognitive changes, supporting performance in both laboratory and everyday settings [12,13]. Although these processes do not eliminate the deficits associated with aging, they partially offset their impact. In this sense, age-related vWM impairments are best understood within a broader framework in which decline and adaptation coexist [3,10].

At the neural level, this adaptive capacity is reflected in the functional reorganization of the brain. In particular, older adults typically show increased engagement of prefrontal control regions alongside reduced recruitment of posterior sensory areas, a pattern described as the Posterior–Anterior Shift in Aging (PASA) [14]. Similarly, the Hemispheric Asymmetry Reduction in Older Adults (HAROLD) model proposes that greater bilateral prefrontal activation during cognitive tasks may support performance through the recruitment of additional neural resources [15]. Extending this perspective, the revised Scaffolding Theory of Aging and Cognition (STAC-r) suggests that the aging brain continuously develops and recruits alternative neural pathways through experience, cognitive engagement, and other life-course factors to partially counteract age-related structural and functional decline [13]. Importantly, increased prefrontal or bilateral recruitment should not, by itself, be interpreted as evidence of successful compensation. Such patterns may reflect compensatory processes when they are associated with preserved or improved behavioral performance, but in the absence of corresponding behavioral benefits they may alternatively reflect greater task demands, reduced neural specificity, or other forms of age-related functional reorganization. Although these models are not specific to vWM, they provide a useful framework for understanding how age-related neural changes may be accompanied by the recruitment of additional control-related resources and alterative cognitive strategies that support the maintenance and regulation of visual information. Collectively, they suggest that a considerable degree of plasticity is retained throughout healthy aging, despite age-related structural and functional changes. This residual plasticity may provide opportunities to support or enhance vWM through targeted interventions in later life. Consistent with this view, growing evidence has examined whether cognitive training, non-invasive brain stimulation (NIBS), physical exercise, and engagement in cognitively and socially stimulating activities can modulate the neural and cognitive processes underlying age-related cognitive functioning [16,17]. The current article presents a narrative review of these approaches, with a particular focus on their potential to support vWM in healthy older adults.

The literature was identified through searches of Google Scholar, PubMed, and Scopus conducted between September 2025 and March 2026. Search strategies combined terms related to aging (e.g., “older adults,” “healthy aging,” “aging”) and visual working memory (e.g., “visual working memory,” “visuospatial working memory”) with terms referring to the main intervention approaches considered in this review, including “cognitive training,” “video game,” “non-invasive brain stimulation,” “NIBS,” “tDCS,” “tACS,” “TMS,” “rTMS,” “tRNS,” “physical exercise,” “metacognition,” “metacognitive strategies,” “combined intervention,” and “virtual reality.” Reference lists of articles and reviews were also examined to identify additional studies of particular relevance.

Studies were considered for inclusion when they involved healthy older adults and either (i) directly trained visual or visuospatial WM or (ii) implemented interventions targeting other cognitive domains but assessed transfer effects on visual or visuospatial WM outcomes. This approach allowed us to consider both the direct effects of interventions specifically targeting vWM and evidence of transfer to vWM following interventions that did not directly train this domain. Within these intervention categories, particular attention was given to studies that were especially relevant to the aims of the review and that provided evidence regarding intervention efficacy, transfer effects, and the methodological or individual factors potentially contributing to variability in outcomes. Given the narrative nature of the review, no formal risk-of-bias assessment, quantitative synthesis, or meta-analysis was performed.

### 2.1. Cognitive Training

One prominent line of research has focused on cognitive training, namely structured interventions designed to enhance specific cognitive abilities through sustained engagement in targeted tasks [18,19]. Traditionally, vWM has been studied through task repetition, which forms the basis of most cognitive training programs. The underlying principle is straightforward: performance improves with practice, a notion deeply rooted in the psychology of learning [20]. Repeated engagement with demanding cognitive tasks, such as n-back or change detection paradigms, is thought to strengthen the neural and cognitive processes that support WM and cognitive control [21]. Task repetition remains one of the most widely used approaches in cognitive training interventions, including those targeting older adults, where it has been extensively employed to maintain or enhance WM and broader cognitive functioning. For example, Li et al. [22] investigated the effects of 45 days of a visuospatial 2-back training in both younger and older adults. Participants practiced the task daily for 15 min, under both a standard condition and a more demanding condition requiring continuous mental shifting and updating of spatial locations. Following training, both age groups showed improvements on the trained tasks, as well as near-transfer effects to untrained WM tasks, including more demanding spatial 3-back tasks and numerical n-back paradigms compared to the control group whereas no far-transfer effects were observed on more complex span tasks. These benefits were still evident at a three-month follow-up, although older adults showed some decline relative to immediate post-training performance.

Recent evidence suggests that training efficacy depends less on simple repetition and more on the use of adaptive task designs, in which task difficulty is continuously adjusted according to the individual’s performance level. By maintaining task demands close to an individual’s capacity limits, adaptive paradigms appear to maximize engagement and optimize learning conditions, thereby enhancing training gains [23,24]. An example of this approach was provided by Richmond et al. [25], who trained older adults using an adaptive WM span paradigm combining verbal and visuospatial tasks. During each training trial, participants simultaneously performed a processing task while maintaining letters or spatial locations for subsequent serial recall. Task difficulty was continuously adjusted according to individual performance. Following 20 training sessions over 4–5 weeks, participants in the training group showed greater improvements than the control group in both verbal and visuospatial WM span. These gains were accompanied by near-transfer effects on the Reading Span task and more limited far-transfer effects, including fewer repetitions on the California Verbal Learning Test and self-reported improvements in everyday attentional functioning [25]. Consistent with this view, some studies employing adaptive vWM training paradigms have reported significant improvements in older adults [25,26,27,28,29,30,31]. Interestingly, Cantarella et al. [29] further showed that these benefits emerged regardless of whether training stimuli were emotionally positive or neutral. Several studies reported benefits persisting up to 8–9 months [26,29,31], although longer-term maintenance was not consistently observed, with [28] finding no training advantage over controls at the one-year follow-up.

Beyond gains in trained vWM tasks, an important question concerns whether training effects transfer to untrained cognitive abilities and everyday functioning. Brehmer et al. [27] investigated domain-general adaptive WM training in both younger and older adults using a combination of verbal and visuospatial tasks, with gains maintained at a three-month follow-up. Both age groups showed significant improvements in near-transfer tasks, namely untrained WM measures closely related to the training tasks, such as Digit Span for verbal WM and Span Board for visuospatial WM. Far-transfer effects were more limited: no specific training-related benefits emerged for reasoning, interference control, or episodic memory, as control participants also showed practice-related improvements in these tasks. More robust far-transfer effects were observed for sustained attention, in which both younger and older adults receiving adaptive training improved more than the control groups. In addition, participants in the adaptive training condition reported fewer everyday cognitive difficulties on the Cognitive Failures Questionnaire. However, not all forms of vWM training yield consistent transfer effects, particularly in older adults. For example, Dahlin et al. [30] examined updating-based training and observed that, while younger adults showed significant improvements in vWM updating (e.g., n-back performance), no comparable transfer effects were observed in older adults.

The approaches described above—commonly referred to as process-based cognitive training—primarily aim to enhance performance through repeated practice on tasks that closely resemble the trained ability, typically targeting either vWM capacity itself or specific vWM processes such as information updating and maintenance. Another line of WM training is represented by structured theory-based WM programs, which are grounded in cognitive models and aim to train broader underlying WM mechanisms rather than task performance itself. An example is the Working Memory training program (WOME; Weicker et al. [32]), which differs from process-based WM training in both theoretical orientation and methodological design. Whereas studies such as Buschkuehl et al. [28], Borella et al. [26], Cantarella et al. [29], Richmond et al. [25], and Zinke et al. [31], used process-based paradigms that directly trained vWM through adaptive n-back, matrix, or change-detection tasks, WOME is an intervention based on Baddeley’s WM model and designed to target storage, inhibition, and manipulation processes. WOME employed a double-blind, placebo-controlled design with an active control group, providing a control over expectancy and motivational effects. In contrast, gains following WOME appeared less durable, although the intervention was associated with improvements in some aspects of everyday functioning.

Taken together, cognitive training can improve vWM performance in older adults, but magnitude and generalizability of these benefits vary across interventions and outcomes. Differences between process-based and theory-driven approaches further suggest that improving task performance and modifying broader cognitive mechanisms may represent distinct challenges. These findings highlight the need for interventions that can achieve broader and more durable improvements in vWM functioning in older adults.

#### Game-Based Cognitive Training

Game-based cognitive training refers to computerized interventions in which participants repeatedly engage with purpose-designed interactive tasks or games over multiple training sessions to stimulate cognitive processes. These interventions may be delivered through platforms for video game training or through immersive technologies such as virtual reality (VR). Video game training can be broadly classified into action and non-action games. Action video games are typically characterized by fast-paced, dynamic, and unpredictable environments that require rapid visuomotor responses, divided attention, and continuous monitoring of multiple stimuli, whereas non-action video games generally involve slower-paced gameplay with lower motor demands and focus on cognitive processes such as memory, attention, reasoning, and problem solving [33]. Evidence from younger adults has primarily been obtained using action video games. For example, Blacker et al. [11] demonstrated that action video game training improved vWM performance, suggesting that repeated exposure to highly demanding visual environments may enhance the encoding, maintenance, and updating of visual information. In contrast, the intervention studies conducted by Ballesteros et al. [34], Toril et al. [33], and Ballesteros et al. [35] used non-action video games, as these are considered more suitable for older adults.

In this context, Ballesteros et al. [34] investigated whether training older adults with non-action video games could enhance cognitive functions that typically decline with age, including visuospatial WM. The intervention was based on Lumosity, a game-based cognitive training platform, and consisted of 20 one-hour sessions over 10–12 weeks. The program included a wide range of computerized cognitive games targeting multiple cognitive functions, several of which specifically engaged WM processes, including Memory Matrix and Rotation Matrix (requiring the encoding and manipulation of spatial patterns), Face Memory (an n-back task involving faces), and Memory Match (comparison between current and previous stimuli). To assess transfer effects to visuospatial WM, the authors employed two tasks: a computerized Corsi Blocks Spatial Span task, and a Jigsaw-puzzle task, requiring the mental reconstruction of fragmented images. Although participants showed clear improvements across training sessions in the games practiced, these gains did not generalize to the outcome measures. Specifically, no significant improvements were observed in either the Corsi task or the Jigsaw task compared to the control group, leading the authors to conclude that this type of non-action video game training could be ineffective in enhancing visuospatial WM capacity in older adults.

In contrast, Toril et al. [33] reported more positive results using a similar but more focused non-action video game intervention. In their study, older adults completed 15 one-hour training sessions, also using Lumosity games, but with a reduced set of six games specifically selected to engage WM processes. As in the previous study by Ballesteros et al. [34], visuospatial WM was assessed using the Corsi blocks task and the Jigsaw-puzzle task. Results showed that the trained group significantly improved in both measures compared to controls, with gains in the Jigsaw task maintained at a 3-month follow-up, although improvements in the Corsi task were not sustained over time. The authors proposed that the different outcomes observed compared with Ballesteros et al. [34] could be attributed to several methodological factors. For example, the more focused training protocol may have limited cognitive overload and allowed participants to engage more effectively with the targeted WM processes. The training context may also have contributed to the positive results: previous research has suggested that the participants’ interest and engagement during training could be influenced by the presence or absence of the experimenter [36]. Although the experimenter had also been present in the earlier study by Ballesteros et al. [34], those sessions involved only small groups of two or three participants. In contrast, the present study was carried out with the whole group together in a shared setting, a condition that may have fostered greater motivation, social involvement, and active participation throughout the intervention. Together, these findings suggest that the efficacy of non-action video game-based interventions for improving vWM in older adults depends not only on training duration, but also on the specific cognitive demands of the games, the degree of task specificity, and psychosocial (e.g., motivation and engagement) and contextual factors.

More recently, Ballesteros et al. [35] conducted a randomized controlled trial designed to overcome several methodological weaknesses identified in previous cognitive training studies, particularly the absence of active control conditions and the limited consideration of motivational factors. Older adults assigned to the experimental condition completed training with Lumosity video games, whereas the control group engaged in simulation strategy games for the same number of sessions. Beyond cognitive performance, the study also monitored participants’ motivation, engagement, and expectations across the intervention, allowing the authors to evaluate whether possible improvements could reflect placebo or contextual effects rather than genuine cognitive transfer. The findings showed that, although participants became more accurate in the trained games, the training did not lead to superior improvements in untrained cognitive abilities compared with the active control group. Only a marginal advantage was observed in the n-back task, while both groups improved similarly on the Corsi Blocks task. These parallel gains suggest that the observed benefits were largely non-specific and may reflect repeated practice or general participation effects rather than selective enhancement of visuospatial WM. Moreover, because the two groups reported comparable levels of motivation, engagement, and expectations, the results cannot be explained by differences in involvement during training.

More recently, game-based cognitive interventions for older adults have been extended with VR, which allows cognitive demands to be embedded within interactive and visually rich environments. For example, Szczepocka et al. [37] investigated the effects of a 12-week home-based immersive VR cognitive training program in healthy older adults aged 65–85 years. Participants assigned to the experimental group completed a series of cognitive exercises targeting attention and WM while interacting with virtual objects. In particular, the WM training consisted of an n-back task in which participants were required to select a balloon matching the color of the balloon presented *n* positions earlier. The control group was exposed to the same type of immersive natural environment through a VR headset but did not perform any cognitive exercises. Following training, the experimental group showed greater improvements than the control group in 1-back performance. However, no significant improvement emerged for the broader WM domain assessed with the CNS Vital Signs battery. These findings provide preliminary evidence that immersive VR may represent a promising extension of game-based cognitive training in healthy older adults, while also reinforcing the pattern observed with conventional game-based interventions: benefits may be more evident for specific trained or closely related cognitive processes than for broader measures of WM functioning.

### 2.2. Non-Invasive Brain Stimulation (NIBS)

Another promising approach for supporting vWM in older adults involves NIBS, a set of techniques designed to modulate neural excitability and network dynamics. Transcranial electrical stimulation (tES) refers to a family of methods that delivers weak electrical currents through electrodes placed on the scalp in order to influence brain activity and cognitive functioning. Among these, transcranial direct current stimulation (tDCS) has been the most widely investigated in relation to vWM, and involves the application of a weak low-intensity current to induce shifts in neuronal membrane potentials that subtly modulate cortical excitability, ongoing brain activity, and task performance.

Evidence from studies using visuospatial n-back paradigms remains limited. These studies have frequently targeted the dorsolateral prefrontal cortex (dlPFC)—a key region within the frontoparietal WM network involved in cognitive control and the maintenance and updating of task-relevant information. However, behavioral effects have not been consistently demonstrated across stimulation protocols and cognitive tasks. Several studies have reported no significant improvements in vWM performance following prefrontal stimulation in healthy older adults [38,39,40,41]. These null findings may partly reflect differences in stimulation sites and task demands [40,41], as well as the limited sensitivity of some behavioral measures to detect subtle changes in vWM performance.

Beyond n-back paradigms, studies employing more ecologically relevant vWM tasks point to the importance of network-level targeting. Frontoparietal stimulation protocols appear particularly effective, as they target network-level interactions rather than isolated regions. Specifically, unilateral (right) frontoparietal tDCS—designed to approximate more “youth-like” patterns of functional lateralization—has been shown to enhance vWM accuracy, especially in older adults with lower baseline capacity, whereas bilateral prefrontal stimulation—often interpreted as reflecting compensatory recruitment—and posterior parietal stimulation appear less effective [42].

A consistent feature across studies is variability in responsiveness to stimulation, with individual differences playing an important moderating role. Berryhill and Jones [38] found that offline tDCS improved performance only in older adults with higher levels of education, regardless of stimulation lateralization, suggesting a moderating role of cognitive and neural reserve. Similarly, Johnson et al. [43] identified education level as the strongest predictor of behavioral gains following frontoparietal tDCS in a visual change-detection task, with electrophysiological analyses further revealing that improvements were associated with enhanced theta-band network synchronization across frontoparietal regions, suggesting that tDCS may facilitate vWM by strengthening large-scale oscillatory dynamics supporting the coordination of storage and control processes.

Transcranial alternating current stimulation (tACS) has gained increasing attention as a complementary NIBS technique. Unlike tDCS, which primarily modulates cortical excitability, tACS applies weak sinusoidal electrical currents to entrain ongoing brain rhythms in a frequency- and phase-specific manner, thereby influencing oscillatory dynamics and cognitive performance. This approach is particularly relevant to vWM, which depends on the coordinated activity of distributed cortical networks.

In healthy older adults, Mitterová et al. [44] found that theta-frequency tACS applied over frontal or frontoparietal regions during n-back task performance enhanced vWM in a load- and montage-dependent manner. Frontal stimulation selectively improved accuracy and RTs under higher vWM load (3-back), whereas in-phase frontoparietal stimulation primarily enhanced accuracy under lower load (2-back), without affecting RTs. These findings suggest that the efficacy of theta-tACS may depend not only on the stimulated regions but also on the cognitive demands imposed on the vWM system. Furthermore, Reinhart and Nguyen [45] showed that age-related vWM decline was associated with reduced theta-gamma phase-amplitude coupling within temporal cortex and reduced theta synchronization between prefrontal and temporal regions. Older adults also lacked the directional prefrontal-to-temporal information flow observed in younger individuals, consistent with an age-related disruption of coordinated communication within the vWM network. Individualized in-phase frontotemporal high-definition tACS increased these patterns of neural synchronization and improved vWM accuracy, with behavioral benefits persisting for at least 50 min after stimulation. Importantly, follow-up experiments supported the anatomical, frequency, and phase specificity of these effects, with antiphase stimulation producing the opposite behavioral effect in younger adults. Together, these findings suggest that impaired synchronization of neural activity within distributed cortical networks may contribute to age-related vWM decline and that appropriately tuned tACS may transiently restore more functional patterns of neural communication.

Complementary evidence indicates that modulating oscillatory activity can also influence inhibitory control mechanisms within vWM: Borghini and colleagues [46] found that parietal alpha-tACS selectively improved resistance to interference from misleading cues in a visuospatial retro-cueing task, suggesting that frequency-specific stimulation may enhance vWM not only by modulating processes related to the maintenance of visual information, but also by improving the selective filtering of competing representations—a process known to decline with age, as discussed in the Introduction. Nevertheless, several limitations should be considered when interpreting these findings. Reinhart and Nguyen [45] employed a highly spatially and spectrally specific stimulation protocol: improvements in vWM were observed when prefrontal and temporal regions were stimulated concurrently using in-phase high-definition tACS individually tuned to each participant’s peak theta synchronization frequency, whereas stimulation of either region alone or frontotemporal stimulation at a fixed, non-individualized frequency did not produce comparable benefits. Although this specificity strengthens the proposed mechanistic link between frontotemporal theta synchronization and vWM performance, it may limit the generalizability and practical applicability of the findings, as the observed effects appear to depend on precise anatomical targeting and individualized frequency calibration. Mitterová et al. [44], in turn, should be regarded as a proof-of-concept study designed to characterize the immediate effects of different theta-tACS montages rather than to establish the durability of cognitive enhancement. The study employed a single stimulation session for each condition, and the clearest benefits emerged during stimulation, whereas evidence for immediate offline transfer was limited. Thus, it remains unclear whether the observed improvements would persist beyond the immediate experimental period, generalize to untrained vWM tasks, or become more robust following repeated stimulation sessions. Importantly, the effects were also heterogeneous across stimulation montages and cognitive loads: frontal and frontoparietal stimulation produced different benefits depending on vWM load. Together, these findings suggest that, despite the mechanistic promise of oscillation-based stimulation, its effects may be highly dependent on stimulation parameters, task demands, and individual characteristics, and larger multi-session studies are therefore needed to establish their robustness, generalizability, and long-term durability.

Compared to electrical stimulation techniques, evidence from transcranial magnetic stimulation (TMS)—which delivers brief magnetic pulses to transiently modulate cortical excitability—remains limited and heterogeneous. Yamanaka et al. [47] provided evidence that right parietal TMS during a delayed match-to-sample task in the visual sensory modality did not significantly alter accuracy but was associated with RT changes and age-dependent differences in cortical activation, with older adults showing reduced hemodynamic activity and weaker propagation of effects to frontal regions, suggesting altered frontoparietal dynamics in aging.

Taken together, the current literature indicates that NIBS can modulate neural systems underlying vWM in older adults, but its behavioral efficacy remains variable and appears to depend on the interaction between stimulation parameters, task demands, and individual characteristics. Specifically, stimulation site and montage, frequency and phase where applicable, cognitive load, baseline performance, and cognitive reserve may all contribute to determining whether and to what extent cognitive benefits emerge. Additionally, the timing and dosing of stimulation represent key unresolved issues: single-session protocols often yield modest and inconsistent effects, whereas repeated or longitudinal interventions show greater promise for more stable cognitive benefits. Considerable variability in electrode placement across tES protocols further complicates interpretation, as stimulation may inadvertently influence interconnected regions and alter network dynamics in unintended ways. Overall, while NIBS represents a promising tool for modulating vWM-related processes in aging, current evidence underscores the need for more systematic and theory-driven approaches, particularly integrating stimulation protocols with task demands and individual cognitive profiles.

### 2.3. Combining Different Methods

An emerging line of research has explored whether combining different intervention approaches may produce synergistic benefits for WM in older adults. Combining different methods represents a promising but complex approach to enhance vWM in aging, with outcomes influenced by both intervention design and individual variability.

#### 2.3.1. Cognitive Training Combined with Physical Exercise

A multimodal approach is grounded in the idea that different interventions may target complementary mechanisms, such as the combination of cognitive training and physical exercise. While physical exercise is associated with neurobiological changes such as increased brain-derived neurotrophic factor (BDNF) and improved neural plasticity [48], cognitive training is thought to enhance specific cognitive processes and strengthen neural efficiency [49].

Nishiguchi et al. [50] investigated the effects of a 12-week multimodal intervention combining physical exercise and cognitive dual-task training in community-dwelling older adults, who were randomly assigned to either an exercise group or a control group. Participants in the exercise group attended weekly 90-min group sessions including strength training and cognitively demanding motor activities requiring the simultaneous execution of physical and cognitive tasks, such as verbal fluency exercises, numerical-response activities, and coordinated stepping tasks, together with a home-based walking program. To examine vWM processes, the authors employed a n-back paradigm during fMRI acquisition. Participants completed 0-back and 1-back tasks involving either spatial locations or face stimuli, requiring continuous monitoring and updating of visual information. Behaviorally, the intervention did not lead to significant improvements in task accuracy or RTs on vWM tasks, which the authors attributed to possible ceiling effects given the cognitively healthy status of the sample. Nevertheless, neuroimaging analyses revealed decreased activation in several regions associated with visual memory processing, particularly within bilateral prefrontal areas, after the intervention. The authors interpreted this reduction in activation as potentially reflecting enhanced neural efficiency, whereby participants may require fewer neural resources to maintain comparable performance levels following training. However, because these neural changes were not accompanied by significant improvements in vWM accuracy or RTs, they should not be considered direct evidence of enhanced neural efficiency or successful compensation. The observed reductions in prefrontal activation did not survive correction for multiple comparisons, and the study did not include separate physical-only or cognitive-only intervention groups, making it difficult to determine which component of the multimodal program primarily contributed to the reported neural changes.

Similarly, Maillot et al. [51] investigated whether exergame training (i.e., interactive video games combining cognitive engagement with physically simulated movements and exercise) could improve cognitive and physical functioning in older adults. Participants completed a 12-week intervention based on Nintendo Wii activities involving simulated sports such as tennis, boxing, balance, and coordination games. Cognitive performance was assessed before and after training through a broad neuropsychological battery targeting executive functions, processing speed, and visuospatial abilities. Visuospatial functioning was evaluated using tasks including the Spatial Span test, in which participants reproduced sequences of spatial locations either in the same order (forward condition) or in reverse order (backward condition), thereby assessing visuospatial short-term and WM abilities. Although the training group showed significant improvements in cognitive control and processing speed measures, no significant transfer effects were observed for visuospatial tasks, including Spatial Span performance. The authors therefore concluded that exergame could benefit executive and speed-related cognitive processes, while producing limited effects on visuospatial WM abilities.

Together, these findings suggest that cognitive training combined with physical interventions may enhance broader executive efficiency in older adults, even though they do not yield consistent benefits specifically for visuospatial WM.

#### 2.3.2. Cognitive Training Combined with NIBS

Combining cognitive training with NIBS, particularly tDCS, has been proposed as a promising approach to enhance vWM in older adults. However, evidence remains mixed and suggests that the efficacy of such combined interventions critically depends on stimulation parameters, neural targeting, and individual characteristics—paralleling findings from NIBS-alone studies.

Several studies indicate that pairing tDCS with WM training does not necessarily enhance performance on trained tasks but may facilitate transfer effects. Stephens and Berryhill [52] paired anodal tDCS over the right dlPFC with multi-component WM training protocol that included both verbal (Operation Span, Subtract-2 Span) and visual WM (visual and spatial recall) tasks. Although all groups improved on trained tasks, only participants receiving the highest stimulation intensity showed significant far-transfer gains to untrained laboratory and everyday tasks at one-month follow-up, suggesting that stronger prefrontal stimulation may promote more durable and ecologically relevant benefits. Similarly, Jones et al. [53] combined multi-session tDCS with a visuospatial WM training protocol comprising visual and spatial recognition and recall tasks, comparing stimulation over the right dlPFC, left posterior parietal cortex (PPC), alternating dlPFC-PPC across sessions, and sham. Improvements on transfer measures—including spatial 2-back task, digit span, and Stroop task—persisted at follow-up only in the active stimulation groups, whereas gains in the sham group were not maintained, pointing to a specific role of frontoparietal stimulation in consolidating visuospatial WM benefits.

In contrast, other studies have failed to observe additive benefits of tDCS over cognitive training alone. Nilsson et al. [54], using anodal tDCS over the left dlPFC combined with a multi-component cognitive training (including task-switching, rule-switching, n-back, and running span tasks), found no significant differences between active and sham groups on post-training assessment or n-back performance. Au et al. [55], who paired left dlPFC stimulation with a training protocol including visual n-back and word list learning tasks, similarly found comparable improvements across active and sham groups on untrained measures of WM and memory, including a visual n-back outcome measure. These null findings are notable in that they extend to visually based WM measures, suggesting that left dlPFC stimulation may not reliably augment training-related gains even when visual WM is part of both training and assessment protocol. Teixeira-Santos et al. [56] adopted a dual n-back training paradigm combined with anodal tDCS over the dlPFC and found training-related gains in both active and sham conditions on the trained task; however, some transfer effects—specifically to reasoning (Raven’s APM) and short-term memory (digit span)—emerged only in the active stimulation group at follow-up, suggesting that tDCS may not consistently enhance online training performance but could selectively influence consolidation or transfer processes under specific conditions.

Importantly, converging evidence highlights a key role of individual differences—particularly baseline WM capacity and age subgroup—mirroring patterns observed in NIBS-alone studies. Assecondi et al. [57] combined anodal tDCS over the right dlPFC with an adaptive spatial n-back training and assessed outcomes using a composite WM score derived from five tasks, including change detection, spatial and visual n-back, and digit span. Results showed that the combined intervention preferentially benefited “old-old” adults with lower initial WM capacity, whereas younger-old participants performed better under sham stimulation, suggesting that individuals with fewer cognitive resources may derive greater benefit from neuromodulation, in line with compensatory accounts.

The effectiveness of combined interventions also appears to depend on stimulation targeting and network engagement. Nissim et al. [58] showed that anodal tDCS over the right dlPFC—explicitly targeting the frontoparietal WM network—combined with multimodal cognitive training (including both WM, one of which targeted vWM, and processing speed tasks) increased functional connectivity within this network and resulted in a near transfer effect on an untrained verbal 2-back task. These findings, suggesting that network-level modulation may underlie behavioral gains, are consistent with findings from NIBS-alone protocols. However, specific effects on the trained tasks (including the visual n-back task) were not reported.

Finally, evidence from other stimulation modalities further supports a nuanced view. Brambilla et al. [59] combined bilateral dlPFC transcranial random noise stimulation (tRNS)—a weak electrical current with randomly varying amplitude and frequency applied through scalp electrodes, generating a noise-like electrical signal that can modulate cortical excitability—with a multi-domain cognitive training protocol. The latter included a spatial WM precision task alongside attention, verbal fluency, and reasoning measures. Despite this broader training design, no improvements in visuospatial WM performance were observed in the active stimulation group relative to sham, indicating that not all forms of non-invasive electrical stimulation yield comparable benefits, and that multi-domain training not specifically targeting vWM may further limit the sensitivity to detect stimulation-related gains on vWM outcomes.

## 3. Towards Optimizing Cognitive Interventions: The Potential of Placebo and Nocebo Effects

Overall, the evidence reviewed indicates that healthy aging is accompanied by a substantial, albeit constrained, capacity for plasticity. Across the studies examined, a range of intervention approaches were shown to modulate the mechanisms supporting vWM, indicating that age-related decline is not fixed but remains responsive to targeted interventions. Cognitive training has shown behavioral benefits mainly on trained and closely related tasks [22,25,26,27,28,29,30,31,60], with some benefits persisting for 3–9 months [22,26,27,29,31]. On the other hand, game-based interventions show heterogeneous outcomes. While Ballesteros et al. [34,35] found improvements limited to trained games and no transfer in vWM outcomes, Toril et al. [33] and Szczepocka et al. [37] reported transfer to vWM measures, partly maintained at follow-up. NIBS findings are also heterogeneous, with outcomes depending on stimulation parameters, neural targeting, task demands, and individual characteristics (e.g., baseline vWM capacity, education, and cognitive reserve) [38,42,43]. While several studies have reported no overall behavioral improvements in vWM following prefrontal stimulation [38,39,40,41], other findings suggest that frontoparietal targeting [42,43,53] and frequency-specific stimulation [44,46] may be more promising, with some evidence for selective benefits of theta- or alpha-frequency stimulation on vWM performance or specific WM processes. Finally, combined interventions show mixed results. Cognitive training combined with physical exercise has produced no transfer to vWM [50,51], whereas combining cognitive training with NIBS may facilitate transfer or maintenance under specific conditions [52,53,57,58], although additive benefits are not consistently observed [42,54,56,59]. The principal findings and methodological characteristics of the studies considered in this review are summarized in Table 1.

Despite these encouraging findings, the magnitude, durability, and generalizability of intervention effects remain highly variable across studies. This variability likely reflects multiple sources of heterogeneity, including differences in intervention characteristics—such as theoretical rationale, duration, intensity, and outcome measures—as well as participant-related factors including age, baseline cognitive functioning, education, and cognitive reserve. Consequently, direct comparisons across studies remain challenging, and the conditions under which interventions are most effective are still not fully understood. Furthermore, although several studies have demonstrated improvements extending beyond the trained tasks, evidence for robust and long-lasting transfer to everyday cognitive functioning remains limited. Consistent with previous meta-analyses [18,19,61,62], the available literature suggests that many interventions primarily enhance performance on trained or closely related tasks, whereas broader cognitive benefits are observed less consistently.

These limitations suggest that, although plasticity is preserved in later life, current interventions generally produce modest, task-specific benefits while often requiring substantial time, effort, and economic resources. Consequently, an important challenge for future research is not only to establish whether these interventions are effective, but also to determine how their efficacy can be optimized. Achieving this goal may require looking beyond the active components of interventions and considering the broader psychological and contextual factors that may shape individual responsiveness and contribute to the variability in outcomes observed across studies. These factors include, among others, prior experiences, information received about intervention efficacy, motivational and emotional states, social cues, and metacognitive interpretations of one’s own performance [63,64,65,66]. According to current theoretical accounts, the interplay among these contextual, psychological, and interpersonal influences can contribute to the emergence of multifactorial phenomena such as placebo and nocebo effects, which have been studied extensively in pain, motor, and clinical domains but remain surprisingly underexplored in cognitive aging. In this sense, placebo and nocebo effects refer, respectively, to beneficial and adverse changes in symptoms or performance that do not arise from the active components of an intervention, but from these accompanying factors [63,65,67]. Importantly, these effects are not restricted to inert substances (e.g., placebo pills) or sham procedures, but may also accompany effective interventions, indicating that placebo and nocebo effects can manifest in intervention outcomes even when an active treatment is provided [68,69]. For this reason, placebo and nocebo effects have traditionally been regarded as sources of confounds that should be minimized or controlled in intervention research. However, they may also help explain why some individuals derive greater benefits than others from the same protocol [70]. If participants’ expectations and other contextual factors contribute to cognitive outcomes [33,36,71], systematically investigating these mechanisms could help to clarify whether and under which conditions placebo and nocebo effects can complement the existing interventions just reviewed and enhance their efficacy.

A key mechanism through which contextual and psychosocial influences are thought to be translated into placebo and nocebo effects are expectations—namely, individuals’ beliefs or predictions regarding the likely outcomes of an intervention [67,72,73]. During any intervention, participants naturally develop expectations about potential benefits or adverse effects, and these expectations can subsequently shape both perceived and objective outcomes [74,75]. Indeed, individuals who anticipate cognitive improvement may exhibit and perceive enhanced performance independently of the intervention, whereas negative expectations or anticipated difficulties may hinder performance or limit intervention benefits.

From a theoretical perspective, several mechanisms provide a plausible basis for expectancy-related modulation of cognition. Expectations can transiently modify psychological states known to influence cognitive performance, including self-efficacy, intrinsic motivation, goal setting, persistence, effort allocation, and arousal [70]. In turn, these processes may affect the degree to which individuals engage with cognitive tasks and interventions, thereby shaping performance even in the absence of changes to the active intervention itself. Consistent with this view, studies have shown that factors such as self-efficacy and perceived stress can predict the magnitude of cognitive improvements following expectancy manipulations [76,77].

A small but emerging body of research has examined placebo and nocebo effects and the eventual role of expectancy across different cognitive domains. Positive expectations have been associated with improvements in perception, memory, problem solving, creativity, WM, and implicit learning, whereas other studies have reported null effects or benefits restricted to subjective evaluations of performance [70]. Overall, however, the available evidence remains mixed. This inconsistency could reflect substantial methodological heterogeneity, including differences in the way expectations are induced (e.g., verbal suggestion, associative learning, recruitment procedures), the cognitive domains assessed, the outcome measures employed, and whether expectations are manipulated directly or merely measured [70].

Importantly, these limitations become even more evident in healthy aging, where empirical evidence remains remarkably scarce [78]. To date, only a handful of studies have directly examined placebo and nocebo effects and the specific role of expectations in older adults. In Oken et al. [76], healthy older adults were randomly assigned to receive either a placebo pill presented as a cognitive enhancer or no pill for two weeks. Participants in the placebo condition showed improvements in delayed recall and choice RT, although these effects were not observed consistently across cognitive domains, and later analyses raised concerns about the robustness of the findings [79]. More recently, a three-week intervention in healthy older adults compared open-label placebos (OPLs), in which participants were informed that the pills they were receiving were inert but could still elicit beneficial mind–body responses, with deceptive placebos, in which participants were led to believe that they were receiving a multivitamin supplement able to enhance functioning, and a no-treatment control [80]. Both placebo groups showed pre-to-post improvements in both cognitive tests, assessing short-term verbal and working memory through Digit Span and selective attention through Stroop task, whereas no significant pre-to-post changes were observed in the control group. Interestingly, OPLs were not inferior to deceptive placebos and also produced significant between-group benefits over controls on Digit Span performance at post. However, treatment expectations were assessed only informally, making it difficult to determine the extent to which the observed effects were related to expectancy-driven mechanisms or other factors. Moreover, despite the population being investigated, no cognitive screening was conducted, preventing confirmation that participants met criteria for cognitively healthy aging. Finally, because pre-to-post changes were analyzed separately within each group, the absence of a significant change in the control group does not by itself rule out practice effects. Moving from placebo administration to the direct manipulation of expectations within a cognitive intervention, Rabipour et al. [81] examined whether expectation manipulation could influence the outcomes of a five-week adaptive computerized cognitive training in a self-selected sample of high-functioning older adults. The multimodal adaptive program, targeting WM, sustained and divided attention, inhibition, and visuospatial perception, was compared with an active control condition involving non-adaptive Sudoku and single-domain n-back exercises. Participants in both conditions were additionally assigned to high- or low-expectation subgroups through brief written suggestions indicating either that the training was known to improve cognitive performance or that its benefits had not been established. At the level of subjective expectations regarding the anticipated effectiveness of the training across different cognitive domains, participants were generally optimistic at baseline, but the suggestive manipulation produced asymmetric short-term changes: immediately after written suggestion, high-expectation participants increased their ratings across all cognitive domains compared to baseline, whereas low-expectation participants significantly reduced their ratings only for general cognitive function. These differences were no longer evident following the five-week intervention, when expectation ratings tended to converge toward more neutral levels, possibly reflecting the limited persistence of the suggestive manipulation or participants’ reassessment of its effectiveness based on their own experiences and perceived performance. At the objective level, participants improved over time on neuropsychological and psychosocial transfer measures, but neither the computerized cognitive training nor written suggestions produced greater improvements than their respective control conditions, and no interaction between training and expectation condition emerged. However, no explicitly neutral-expectation condition was included, and participants’ high baseline expectations may have limited the scope for expectation modulation. Overall, the study provides no evidence that brief written suggestion enhanced cognitive transfer, although the limited and asymmetric manipulation of expectations leaves open the possibility that stronger or differently delivered expectation manipulations might yield different effects.

Beyond intervention studies, recent experimental work has also begun to disentangle the specific contribution of verbal suggestion from the broader psychosocial context typically accompanying placebo and nocebo effects. To this end, Barbon et al. [82] experimentally manipulated positive and negative written suggestions regarding the efficacy of an inert intervention before healthy older people completed an online perceptual-attentional task. Since the study was conducted remotely, participant–experimenter interactions and other contextual influences that may contribute to expectancy effects in face-to-face settings—including interpersonal cues, demand characteristics, and socially driven response biases—were minimized [83], allowing the impact of written suggestions on cognitive performance to be assessed more directly. Whereas negative suggestions impaired objective performance despite no corresponding change in subjective expectations and perceived intervention efficacy, participants receiving positive suggestions expected improvements and perceived benefits without improving objective performance. These findings suggest that placebo- and nocebo-related influences on cognition may dissociate at the subjective and behavioral levels, highlighting the complexity of expectancy mechanisms and the likely contribution of additional contextual, motivational, interpersonal, and individual factors that may either facilitate or constrain the emergence of measurable objective or subjective effects.

Taken together, the available evidence does not yet allow firm conclusions regarding whether, to what extent, or under which conditions placebo and nocebo effects can manifest in objective cognitive performance and subjective beliefs in older adults. Importantly, the four studies discussed above are not directly comparable, as they differ substantially in the nature and delivery of the expectancy manipulation, the intervention context, the control conditions, the cognitive outcomes assessed, and the timing of outcome measurement.

While Oken et al. [76] and Barbiani et al. [80] involved placebo pills administration over several weeks, Rabipour et al. [81] embedded a brief written expectation manipulation within a multi-week cognitive training intervention, and Barbon et al. [82] examined the effects of positive and negative written suggestions in a single-session, remotely administered paradigm. These differences should therefore be taken into account when interpreting the apparent convergence and divergence of their findings. Importantly, the available evidence does not support a uniform pattern. Oken et al. [76] and Barbiani et al. [80] reported some improvements in objective cognitive performance that were compatible with placebo-related effects, although the interpretation of these findings is constrained by the methodological limitations discussed above. In contrast, Rabipour et al. [81] and Barbon et al. [82] showed that changes in subjective expectations following written suggestion did not necessarily translate into measurable improvements in objective cognitive performance. The latter two studies primarily addressed the role of experimentally manipulated expectations rather than the broader constellation of processes underlying placebo and nocebo effects.

Overall, the available findings suggest that placebo and nocebo effects can be observed in older adults, but that their expression is not consistent across subjective and objective outcomes. Importantly, the current evidence does not indicate that placebo effects on objective cognitive functioning are absent, but rather that their occurrence appears variable and cannot be readily inferred from changes in subjective expectations alone. This issue is particularly relevant in healthy cognitive aging, as evidence from younger populations or from other domains, such as pain management, may not fully capture how expectancy effects operate in older adults, whose beliefs about their cognitive abilities and potential for improvement may differ from those of younger individuals [84]. Basic experimental research specifically investigating placebo and nocebo mechanisms in healthy cognitive aging therefore remains particularly warranted. An important step will be to establish experimental designs capable of distinguishing placebo and nocebo influences from changes arising from repeated testing, and other factors. Repeated pre–post assessment in an experimental group alone cannot establish placebo and nocebo effects, as observed changes may reflect practice or habituation to the outcome measures. Including a control group that undergoes the same assessments and procedures, but does not receive the experimental manipulation, provides a first means of addressing this concern: changes that do not significantly differ between groups are consistent with practice or other effects unrelated to the manipulation, whereas a greater pre–post improvement or deterioration in the experimental condition would provide evidence that the manipulation contributed to the outcome. Ideally, this pre-post difference should be tested through a between-group comparison of change over time rather than by relying on significant within-group pre–post changes alone. However, even a significant between-group difference does not by itself establish the specific mechanisms underlying a placebo or nocebo effect. Stronger designs should therefore combine appropriate control conditions with direct assessment of expectations and, where possible, measures of motivation, engagement, effort, mood, and other theoretically relevant factors that may influence cognitive performance depending on the population, intervention, and outcome under investigation. Such an approach would allow researchers not only to distinguish placebo and nocebo effects from practice effects, but also to investigate the broader range of mechanisms that may contribute to their manifestation.

Once a solid experimental framework is reached, a further step will investigate whether placebo- and nocebo-related mechanisms can contribute to measurable changes in cognitive performance and whether they might eventually be leveraged to complement cognitive interventions on vWM in older adults. Preliminary evidence from younger adults suggests that such effects can manifest in objective cognitive outcomes [32,74,75,85], including vWM. Hooyman et al. [86] compared a no-exposure control group with sham and active tDCS during training on an adapted Corsi Blocks Task. The sham and active tDCS groups, considered together, showed greater improvement across training than the no-exposure control group, a pattern interpreted as consistent with a placebo effect of tDCS. Moreover, participants’ expectations regarding tDCS efficacy and their belief that they had received active stimulation were associated with the magnitude of this effect. Zhao et al. [87], combining oxytocin versus placebo with neutral, positive, or negative information about the expected effects of the oxytocin, the authors found no main effects of treatment or expectancy induction on WM accuracy, but significant interactions between the two factors across verbal, spatial, and social n-back tasks. Specifically, oxytocin was associated with both expectancy-induced enhancement and impairment of WM performance. Although these findings provide preliminary evidence that placebo- and nocebo-related processes may influence objective WM performance, they derive from younger populations and therefore cannot be assumed to generalize to healthy cognitive aging. Future studies should therefore directly compare expectancy-based and established training approaches not only in terms of cognitive efficacy and transfer, but also with respect to durability, participant burden, intervention intensity, and economic costs. Moreover, this line of research should build on the existing evidence by refining methodological approaches to the study of placebo and nocebo effects in cognitive aging, including through experimentally induced expectations and other manipulations that may require deception. Given the potential ethical implications of such procedures, their scientific value should be carefully balanced against the risk of adverse effects and accompanied by appropriate ethical oversight and post-experimental debriefing. At the same time, non-deceptive approaches such as open-label placebo paradigms may provide a complementary avenue for investigation, particularly when the use of deception is not appropriate or cannot be ethically justified. Preliminary findings from Barbiani et al. [80] suggest that this approach warrants further investigation in older adults, although the methodological limitations of the study preclude firm conclusions regarding its efficacy or underlying mechanisms. Once the mechanisms and boundary conditions of placebo- and nocebo-related effects in cognitive aging are better understood, future research could examine whether similar effects can be elicited through transparent, non-deceptive approaches and whether their underlying mechanisms differ from those involved in deceptive placebo paradigms.

## 4. Conclusions

Healthy aging is accompanied by declines in vWM, yet the evidence reviewed indicates that these changes occur alongside a relatively preserved capacity for plasticity. Cognitive training, game-based approaches, NIBS, and multimodal interventions may improve specific aspects of vWM, although their behavioral effects remain heterogeneous and are often modest, task-specific, and influenced by considerable individual variability. These findings suggest that supporting cognitive aging may require moving beyond the search for a single optimal intervention toward a framework that considers the interplay of neural, cognitive, psychological, interpersonal, and contextual factors. Within this broader perspective, placebo and nocebo effects represent a promising but little-explored area of research in healthy cognitive aging. Rather than being viewed solely as potential confounds, they may provide the framework for investigating how contextual and psychosocial factors contribute to changes in vWM, particularly in older adults. Future research should therefore combine rigorous experimental designs with mechanistic investigations to distinguish placebo and nocebo effects from practice and other non-specific changes, identify the factors that facilitate or constrain their emergence, and determine whether such mechanisms can ultimately be used to complement established interventions.

## Figures and Tables

**Table 1:** Summary of the studies included in the review on approaches to supporting vWM in older people.

Cognitive Intervention	Study	Sample	Intervention Protocol	vWM Tasks Assessed	vWM Task Time Point Assessment	Main Findings Related with vWM
**Cognitive** **training**	Borella et al. (2014) [26]	Experimental group: 20 young-old (M = 69.90, SD = 2.79); 20 old-old (M = 79.60, SD = 2.28). Active control group: 27 young-old (M = 69.55, SD = 2.89); 20 Old-old (M = 79.70, SD = 2.3).	Adaptive training: 3 sessions × 60 min × 2 weeks; 2-day interval between training sessions.	Trained task = adaptive Matrix task: participants need to simultaneously process and recall dot locations in 4 × 4 matrices. Outcome measure = Matrix test.	Outcome measure: pre- and post-intervention, 8-month follow-up.	Significant training-related gains in visuospatial WM emerged in both young-old and old-old adults and remained significantly relative to active controls at the 8-month follow-up.
	Brehmer et al. (2012) [27]	Experimental group: 26 OA (M = 63.9, SD = 3.4); 29 YA (M = 26.2, SD = 2.8). Active control group: 19 OA (M = 63.6, SD = 3.1); 26 YA (M = 25.7, SD = 3.5).	Adaptive training: 20–25 session × 26 min × 5 weeks; at least 20 training days.	Outcome measures = Span Board Forward and Backward tasks. Trained task = participants remember the locations of stimuli presented sequentially in a 4 × 4 grid and reproduce them using the computer mouse.	Outcome measures: pre- and post-intervention, 3-month follow-up.	Adaptive WM training produced significant improvements in both tasks compared with the active control, with these benefits maintained at follow-up.
	Buschkuehl et al. (2008) [28]	32 OA (M = 80.1, SD = 3.6): experimental group: 13 OA; active control group: 19 OA.	Adaptive training: 23 sessions × 45 min × 12 weeks (twice a week frequency).	Trained tasks = 3 adaptive WM tasks: participants need to memorize and reproduce sequences of colored squares (Variant 1) and subsequently perform orientation judgments on animal pictures while simultaneously maintaining and reproducing their presentation sequence (Variants 2 and 3). Transfer task = Block-Span Task: participants need to reproduce the spatial sequence of a blue dot by pointing to the corresponding locations.	Transfer task: pre- and post-intervention; 12-month follow-up (11 OA from experimental group, 11 OA from active control group only).	After training, the experimental group showed a significant transfer effect in the Block-Span task performance relative to controls. The significant vWM improvement was no longer evident at follow-up.
	Cantarella et al. (2021) [29]	Exp 1: experimental group: 18 OA (M = 68.11, SD = 3.16); active control group: 17 OA (M = 68.82, SD = 2.92). Exp 2: experimental group: 16 OA (M = 68.18, SD = 3.63); active control group: 19 OA (M = 69.31, SD = 3.23).	Adaptive training: 3 sessions × 60 min × 2 weeks; 2-day interval between training sessions.	Trained task (and outcome measure) = Image Matrix Task: participants need to maintain the spatial positions of the images displayed on 4 × 4 matrix while simultaneously pressing the spacebar whenever an image appeared in a grey cell. In Exp 2, Image Matrix Task with positive pictures only.	Trained task (and outcome measure) = pre- and post-intervention, 8-month follow-up.	Across both experiments, the experimental groups showed significant vWM improvements compared with the controls. These training-related gains remained evident at follow-up, although decline from post-test was observed in Exp 2.
	Dahlin et al. (2008) [30]	Experimental group: 16 OA (M = 68.38, SD = 1.66); 17 YA (M = 23.67, SD = 2.92). Passive control group: 16 OA (M = 68.25, SD = 1.73); 15 YA (M = 24.09, SD = 2.12).	Adaptive training: 15 sessions × 5 weeks × 45 min.	Trained task = participants were presented with sequences of spatial locations and were required to continuously update the information and recall the last four locations in the correct order.	Pre- and post-intervention.	OA showed training-related improvements from Week 1 to Week 5, with a magnitude of improvement comparable to that observed in YA.
	Li et al. (2008) [22]	Experimental group: 21 OA (mM = 74.5, SD = 2.8); 19 YA (M = 25.3, SD = 2.8). Passive control group: 20 OA (M = 73.3, SD = 2.8); 27 YA (M = 26.4, SD = 2.4).	45 sessions × 15 min/day.	Trained task = spatial 2-back task. Outcome measures = spatial 2-back and 3-back tasks.	Outcome measures: pre- and post-intervention; 3-month follow-up (15 OA, 11 YA only).	Both YA and OA showed substantial improvements in the trained spatial 2-back task, compared with controls. Training effects also transferred to an untrained, more demanding spatial 3-back task: the training group showed significantly greater pre-to-post improvements than controls. At the 3-month follow-up, for the practiced spatial 2-back task, YA maintained their post-training performance better than OA. However, OA still performed substantially better at the 3-month follow-up than pre-intervention. Untrained spatial 3-back task performance was also maintained over the 3-month period in both age groups.
	Richmond et al. (2011) [25]	40 OA (M = 66): experimental group: 21 OA; active control group: 19 OA.	Adaptive training: 20 sessions × 30 min.	Training task = adaptive spatial subtest: participants need to make symmetry judgments about partially filled black-and-white matrices and simultaneously encode and then recall the sequence of highlighted locations presented on a 4 × 4 grid.	Pre- and post-intervention.	In the experimental group, spatial memory span increased significantly from Session 1 to Session 20. No comparison with the active control group as regards vWM performance was reported.
	Weicker et al. (2018) [60]	Experimental group: 20 OA (M = 67.8, SD = 3.9). Active control group: 20 OA (M = 67.7, SD = 3.1). Passive control group: 20 OA (M = 67.5, SD = 5.7).	Adaptive training: 12 sessions × 45 min × 4 weeks; 3 sessions/week.	Trained task = adaptive card-based WOME tasks. The vWM-relevant components required participants to maintain visually presented cards, selectively encode relevant cards while ignoring distractors, and actively manipulate retained visual information. Main outcome measures = Span Board Backward task; Spatial Addition task. Near transfer tasks = Span Board Backward; Spatial Addition task.	Main outcome measures: pre- and post-intervention, 3-month follow-up. Near transfer tasks: post-intervention and 3-month follow-up.	The adaptive WOME group showed a significant pre-to-post improvement on the untrained Span Board Backward task, whereas neither the active nor passive control group showed significant changes. No significant training-specific effect emerged for Spatial Addition. At follow-up, the immediate visuospatial WM benefit was not maintained.
	Zinke et al. (2013) [31]	80 OA (M = 77.2, SD = 8.1): experimental group: 40 OA; passive control group: 40 OA.	Adaptive training: 9 sessions × 3 weeks.	Trained task = Adaptive Picture Grid Task. Outcome measures = Corsi Block Span task; Picture Grid Task: participants memorized common objects and their locations within a grid and subsequently recalled both the objects and their positions.	Outcome measures: pre- and post-intervention, 9-month follow-up.	The experimental group showed significantly greater pre-to-post improvement than the control group on the Picture Grid Task, and this training advantage remained evident at follow-up. In contrast, although performance on the Corsi Block Span task improved from pre- to post-intervention, the improvement did not differ significantly between the experimental and control groups.
**Game-based** **cognitive training**	Ballesteros et al. (2014) [34]	Experimental group: 17 OA (M = 68.8, SD = 5.15). Passive control group: 13 OA (M = 69.2, SD = 5.91).	Lumosity platform with 10 non-action video games: 20 sessions × 1 h × 10/12 weeks.	Trained tasks = Memory Matrix, Rotation Matrix, Face Memory, Speed Match, and Memory Match. Outcome measures = Corsi Blocks task; Jigsaw-Puzzle Task; Rey-Osterrieth Complex Figure Test. Transfer tasks = Corsi Blocks task; Jigsaw-Puzzle Task.	Outcome measures: pre-intervention. Transfer tasks: post-stimulation.	Improvement in all trained tasks but no significant transfer to visuospatial WM outcomes.
	Ballesteros et al. (2017) [35]	Experimental group: 30 OA (M = 66.40, SD = 5.64). Active control group: 25 OA (M = 64.52, SD = 4.51).	Lumosity platform with 10 non-action video games: 16 sessions × 40/50 min × 10/12 weeks.	Outcome measure = Corsi Blocks task. Trained tasks = Tidal Treasures, in which participants selected objects and had to remember their choices; Pinball Recall, which required predicting a ball’s path. Transfer tasks: untrained visuospatial WM task, where sequential black squares were presented at different locations within a 3 × 3 matrix, and participants reproduced the sequence in the same order.	Outcome measure: pre- and post-intervention.	Performance improved significantly across the trained tasks. Performance on the untrained Corsi Blocks task improved significantly from pre- to post-intervention. However, the active control group improved to a similar extent. Therefore, there was no evidence of a training-specific transfer effect to visuospatial WM from the adaptive cognitive games.
	Szczepocka et al. (2024) [37]	Experimental group: 35 OA (M = 69.3, SD = 2.84). Active control group: 37 OA (m = 71.3, SD = 4.2).	Immersive VR-based cognitive training: 36 sessions × 12 min × 12 weeks; at least 3 sessions/week.	Trained tasks = memory module, based on n-back tasks, in which participants had to tap a balloon of the same color as the balloon presented n items earlier; 1-back and dual-n-back exercises. Outcome measures = Part 3 (1-back) of the Four-Part Continuous Performance Test (FPCPT) from the CNS Vital Signs battery.	Outcome measures: pre- and post-intervention.	The experimental group showed significantly greater pre-to-post improvement than the control group on the untrained 1-back task in Part 3.
	Toril et al. (2016) [33]	Experimental group: 19 OA (M = 69.95, SD = 6.73). Control group: 20 OA (M = 73.20, SD = 6.48).	Lumosity platform with 10 non-action video games: 15 sessions × 1 h × 7/8 weeks.	Outcome measures = Corsi Blocks task; Jigsaw-Puzzle Task; Rey-Osterrieth Complex Figure Test. Trained tasks = Memory Matrix; Rotation Matrix; Face Memory; Speed Match; Moneycomb. Transfer tasks = Corsi Blocks task; Jigsaw-Puzzle Task.	Outcome measures: pre-intervention. Transfer tasks: post-stimulation, 3-month follow-up.	Significant training-related improvements were observed for trained tasks, and these training gains transferred to both untrained visuospatial WM measures. At follow-up, the Jigsaw benefit was maintained, whereas the Corsi improvement had declined.
**NIBS**	Arciniega et al. (2018) [42]	Exp 1A: 36 OA (M = 67.72, SD = 4.52). Exp 1B: 36 OA (M = 64.52, SD = 5.41).	Exp 1A: online anodal tDCS, 3 within-subject sessions: unilateral (R) frontoparietal (F6; cathode P6), bilateral frontal (F6; cathode F5), sham; 2 mA current for 20 min. Exp 1B: single-session offline anodal tDCS, 2 conditions: R-PPC (P4; cathode: contralateral cheek), sham; 1.5 mA × 10 min.	Exp 1A: Corsi Blocks Spatial Span task as baseline measure of vWM capacity. Main task: arrays of 3 or 6 colored circles, followed by a delay and a single-probe old/new recognition response. Exp 1B: Automated Operation Span task as baseline vWM capacity. Main task: array of grayscale drawings of common objects on a grid followed by a delay and a single probe item old/new recognition response.	Exp 1A: During stimulation Exp 1B: post-stimulation.	Exp 1A: unilateral frontoparietal stimulation provided greater benefit to vWM than bilateral frontal stimulation. The effects of stimulation montage (unilateral vs. bilateral) varied according to vWM capacity (high vs. low). No post hoc tests reported. Exp 1B: anodal tDCS to PPC did not alter VWM performance.
	Berryhill & Jones (2012) [38]	24 OA (M = 63.7).	Offline anodal tDCS, 3 within-subject sessions: R-dlPFC (F4), L-dlPFC (F3), sham; 1.5 mA current × 10 min; cathode: contralateral cheek.	Offline task = visuospatial 2-back task.	Post-stimulation (no baseline).	No effect of stimulation site on vWM performance. The effects of stimulation site (R- vs. L-dlPFC) × WM task (verbal vs. visual) varied across education groups (high vs. low); post hoc tests were not provided.
	Borghini et al. (2018) [46]	25 YA (M = 24.8, SD = 4.3). 25 OA (M = 69.1, SD = 4.5).	Exp. 1: no stimulation, task performance only. Exp. 2 (OA only): Online bilateral parietal (P3 and P4) tACS, 4 within-subject sessions: 10 Hz (alpha), 4 Hz (theta), 35 Hz (gamma), sham; 1.5 mA current × 20 min. Exp. 3 (OA only): online bilateral parietal tACS; 2 within-subject sessions: 10 Hz (alpha), sham; 1.5 mA current × 20 min.	vWM retro-cueing task: 4 colored/oriented arrows were presented for memorization, followed by a delay period and a valid, invalid, or neutral retro-cue. After a second delay period, participants were asked to recall the orientation of one of the memorized arrows. Exp 3: task-irrelevant vs. neutral retro-cues.	During stimulation.	Exp 1: OA showed poorer inhibitory control than YA, particularly for invalid cues. Exp 2: Alpha-tACS selectively improved target responses and reduced misbinding errors, particularly for invalid cues; theta- and gamma-tACS had no significant effects. vWM recall precision did not significantly change across stimulation conditions. Exp. 3: Alpha-tACS reduced the disruptive effect of task-irrelevant cues on WM performance.
	Firouzkouhi Moghadam et al. (2020) [39]	45 OA (M = 61.9, SD = 2.2).	5-session offline anodal tDCS, 3 between-group conditions: R-dlPFC (F4), L-dlPFC (F3), sham; 2mA current; cathode: contralateral supraorbital area.	Offline task = 2-back task (with figures).	Pre-intervention, before the Section 3, post-intervention, 2 weeks follow-up.	No between-group differences in vWM at any time point (separate analyses per time point). Significant within-group change from pre-intervention only in F3-stimulation. No direct group × time comparison reported.
	Johnson et al. (2022) [43]	30 OA (M = 67.33, SD = 3.57).	Offline anodal tDCS, 3 within-subject sessions: PFC-PPC (F6; cathode: P6), PFC-CC (F6; cathode: contralateral cheek), sham; 2 mA current × 20 min. EEG recorded during task performance.	Corsi Blocks Spatial Span task, together with other WM measures, to assess baseline WM capacity. Main task = visual change-detection task.	WM capacity at pre- and post-stimulation.	By collapsing across stimulation site and set size, tDCS improved vWM accuracy and RT, with greater accuracy improvement in highly educated OA; baseline Corsi vWM capacity did not influence outcomes. Greater vWM accuracy improvement following tDCS was associated with stronger theta-network enhancement, whereas tDCS-related faster RT was associated with greater theta–gamma PAC enhancement.
	Mitterová et al. (2026) [44]	38 OA (M = 68.37, SD = 5.76).	Online theta (4.51 Hz) tACS, 3 within-subject sessions: R-MFG, R-MFG–R-IPL, sham; 1.5 mA peak-to-baseline × 20 min. fMRI-individualized stimulation sites.	Visuospatial 2-back and 3-back tasks.	During stimulation.	Frontal stimulation improved 3-back accuracy & RT. Frontoparietal stimulation improved 2- and 3-back task accuracy. tACS did not improve RT in 2-back task over sham.
	Nilsson et al. (2015) [40]	30 OA (M = 69, SD = 7).	Single-session offline and online anodal tDCS targeting L-dlPFC (F3) × 25 min; 3 within-subject intensity conditions: 1 mA, 2 mA, sham; cathode: contralateral supraorbital area.	Visual 3-back task.	Pre-stimulation (T0), during stimulation at 0–5 min (T1), at 10–15 min (T2) and at 20–25 min (T3), post-stimulation at 5–10 min (T4) and 30–35 min (T5).	No overall difference in vWM performance between groups.
	Reinhart & Nguyen (2019) [45]	42 OA (M = 68.8, SD = 4.4). 42 YA (M = 24.4, SD = 2.8).	Online HD-tACS, within-subject conditions (OA only): frontotemporal theta-tuned (L-PFC–L-TC), sham; 1.6 mA 1.6 mA peak-to-peak × 25 min. YA received sham. EEG recorded during task performance.	Visual change-detection task.	During and up to 50 min post-stimulation.	Compared with YA, OA were less accurate and slower and showed reduced frontotemporal theta synchronization and disrupted PFC to TC information flow under sham. Active HD-tACS increased theta synchronization and restored PFC to TC information flow in OA, alongside improved vWM accuracy, eliminating the baseline age-group difference. Accuracy improvements emerged after ~8–12 min and persisted throughout the post-stimulation period; RT was not significantly improved overall.
	Seo et al. (2011) [41]	24 OA (M = 70, SD = 4.7)	Offline anodal tDCS, 2 between-group conditions: L-dlPFC (F4), sham; 2mA current × 30 min; cathode: L-arm.	Visuospatial 2-back task.	Pre- and post-stimulation.	No overall difference in vWM performance between stimulation and sham groups.
	Yamanaka et al. (2014) [47]	38 OA (M = 72.4, SD = 4.7) 52 YA (M = 23.4, SD = 2.7)	Online TMS; 2 between-group stimulation sites: (L) parietal (P3), (R) parietal (P4); 2 within-subject sessions: active TMS, sham. Stimulation intensity: 100% (R) rMT; 30 pulses at 5 Hz × 10 trains. NIRS recorded during task performance.	Visuospatial WM and control delayed match-to-sample tasks.	During stimulation.	No TMS-related effect on vWM accuracy in either age group. In OA, faster RTs with P3- than P4- stimulation, irrespective of active vs. sham TMS. Oxy-Hb changes were larger in OA than YA across conditions. In YA, active P3- and P4-stimulation produces opposite task-dependent oxy-Hb patterns, whereas in OA, both P3- and P4-stimulation showed decreased oxy-Hb during vWM task and increased oxy-Hb during the control task (as P3-stimulation in YA), with fewer significant channels and less distinct site-specific patterns than in YA.
**Cognitive training combined with physical** **exercise**	Maillot et al. (2012) [51]	Experimental group: 15 OA (M = 73.47, SD = 4.10). Passive control group: 15 OA (M = 73.47, SD = 3.00).	12 weeks × 1/2 h/sessions. Exergame training using Nintendo Wii physically simulated sports, including Wii Tennis, Boxing, Bowling, Soccer Headers, Ski Jump, Marbles, Ski Slalom, Hula Hoop, Trampoline, and Tennis Return of Serve.	Outcome measures = Spatial Span Forward and Backward.	Pre- and post-intervention.	No significant behavioral improvement in visuospatial WM.
	Nishiguchi et al. (2015) [50]	Experimental group: 24 OA (M = 73.0, SD = 4.8). Passive control group: 24 OA (M = 73.5, SD = 5.6).	12 weeks × 90 min/session; daily pedometer-based walking. Each weekly session comprised 15 min of stretching/moderate exercise, 15 min of strength training, and 60 min of dual-task exercise. Task intensity and difficulty were progressively increased over the intervention.	Outcome measures = location 1-back and face 1-back tasks during fMRI.	Pre- and post-intervention.	No behavioral vWM improvement in both tasks. fMRI showed reduced activation in bilateral prefrontal areas, potentially reflecting greater neural efficiency. However, findings did not survive multiple-comparison correction.
**Cognitive training combined with NIBS**	Assecondi et al. (2022) [57]	28 OA (M = 67.9, SD = 6.1); post hoc age groups: young-old and old-old, based on a median age split (69.5 years).	5-session intervention combining vWM training with online anodal tDCS; between-subjects design: active tDCS or sham. Active stimulation: 2 mA to R-dlPFC (F4) × 20 min/session; cathode: contralateral supraorbital area (Fp1).	Trained task = adaptive spatial n-back task. Outcome measures = change detection task, spatial n-back task, and visual n-back task (included in a composite WM capacity score).	Trained task: during stimulation. Outcome measures: pre- and 48 h post-stimulation.	All participants improved across training sessions. Baseline WM capacity predicted training performance, with higher baseline WM capacity associated with better performance. Active tDCS benefit was selective to old-old OA, particularly those with lower baseline WM capacity, during training (rather than on the post-training composite WM capacity) relative to sham, whereas sham outperformed active tDCS in the young-old group. The increase in composite WM capacity from pre- to post-intervention was not modulated by stimulation.
	Au et al. (2022) [55]	55 OA (M = 71.32).	5-session intervention combining multimodal training with online anodal tDCS; between-subjects design: active tDCS × daily training, active tDCS × every-other-day training, sham × daily training, sham × every-other-day training. Active stimulation: 2 mA to L-dlPFC (F3) × 25 min/session; cathode: contralateral supraorbital area.	Trained task = adaptive visual n-back task, with the n-back level continuing adaptively from the previous training session.	Pre- and post-intervention.	Post-intervention visual n-back data were unavailable for 4 active tDCS and 4 sham OA. Across the 5 training sessions and post-intervention assessment, no advantage of active tDCS over sham on trained n-back performance. However, among OA with lower baseline n-back performance, the active tDCS group outperformed the sham group, whereas no active active–sham stimulation difference was observed among OA with higher baseline performance.
	Brambilla et al. (2021) [59]	1 mA tRNS group: 14 OA (M = 69.4, SD = 5.8). 0.705 mA tRNS group: 14 OA (M = 69.2, SD = 8.7). Sham group: 19 OA (M = 69.4, SD = 6.3)	5-session intervention of combining multimodal cognitive training (WM, inhibition, and cognitive flexibility) with online high-frequency tRNS; between-subjects design: 1 mA + cognitive training, 0.705 mA + cognitive training, sham + cognitive training. Active stimulation: bilateral frontal (anode: F3, cathode: F4) 100–640 Hz tRNS × 20 min/session. Online stimulation commenced with cognitive training; cognitive training lasted 30 min.	Trained tasks = gamified adaptive visuospatial n-back tasks Spatial WM precision Outcome measure = visuospatial WM task: 4 colored bars with random orientations were presented for memorization, followed by a delay period and a color-matched probe bar. Participants were then asked to recall the orientation of one of the memorized bars and reproduce it.	Outcome measure: pre-, 48 h post-stimulation, 1-month follow-up	No specific vWM trained-task effects were reported. No visuospatial WM advantage over sham at post-stimulation nor follow-up.
	Jones et al. (2015) [53]	72 OA (M = 64.38, SD = 5.08).	Offline 10-session anodal tDCS, 4 between-group conditions: R-PFC (F4), R-PPC (P4), alternating PFC & PPC, sham; 1.5 mA ×10 min; reference electrode: contralateral cheek.	Trained tasks (post-stimulation) = visual recognition task; visuospatial recognition task; visual recall task; visuospatial recall task. Near transfer task = spatial 2-back task.	post 1st intervention, post last intervention, 1-month follow-up.	Active tDCS groups were collapsed across stimulation site due to no significant site differences. All groups showed practice-related improvements in trained and transfer tasks post-training, but only active tDCS maintained these gains at follow-up. Recall tasks showed greater gains than recognition tasks at follow-up in both active tDCS and sham groups.
	Nilsson et al. (2017) [54]	tDCS + WM group: 32 OA (M = 69.31, SD = 2.73). tDCS + control group: 30 OA (M = 69.87, SD = 2.91). Sham + WM group: 33 OA, (M = 69.64, SD = 2.97). Sham + control group: 28 OA (M = 69.82, SD = 2.62).	~19-session adaptive multi-component WM or perceptual matching speed (i.e., control) training combined with online anodal tDCS or sham. Between-group conditions: tDCS + WM training, tDCS + control training, sham + WM training, sham + control training. Active stimulation: 2 mA to L-dlPFC (F3) × 25 min/session; cathode: contralateral supraorbital area. Two trained tasks performed during stimulation, when active, and two post-stimulation.	Trained tasks = visuospatial and verbal 2-back and 3-back task, visuospatial and verbal 2-last and 3-last running span. Transfer tasks = untrained-stimulus n-back task, untrained-stimulus task switching, untrained-stimulus rule switching, spatial updating task, spatial switching, spatial switching task.	2-week pre-intervention and 1-week post-intervention.	No advantage of active tDCS + WM training over WM training or active tDCS alone on trained or transfer composite measures. No advantage of multi-session tDCS alone across training groups.
	Nissim et al. (2019) [58]	28 OA (M = 73.67, SD = 7.32): 14 active tDCS group (M = 73.57, SD = 7.84); 14 sham group (M = 73.78, SD = 7.06).	10-session intervention combining multimodal (WM and processing-speed tasks) cognitive training with online anodal tDCS; between-subjects design: cognitive training + active tDCS, cognitive training + sham. Active stimulation: bilateral frontal tDCS (anode: R-dlPFC, F4; cathode: L-dlPFC, F3), 2 mA × 20 min/session. Stimulation during the first 20 min of each 40 min training session. fMRI recorded pre- and post-intervention (during verbal 2-back task).	Trained task = visual n-back task.	-	No specific vWM trained-task effects were reported. For the near-transfer effects (i.e., the untrained verbal 2-back task), active tDCS + multimodal cognitive training improved 2-back target accuracy relative to sham + multimodal cognitive training at post-intervention. Active tDCS also increased L-DLPFC–R-IPL functional connectivity during the 2-back task relative to sham; no corresponding connectivity effect was observed during 0-back.
	Stephens & Berryhill (2016) [52]	1 mA tDCS group: 30 OA (M = 68.6). 2 mA tDCS group: 30 OA (M = 68.6). Sham group: 30 OA (M = 69.9).	5-training-session multi-component WM training + online anodal tDCS R-dlPFC (F4) (between-group conditions; 1 mA tDCS, 2 mA tDCS, sham × 15 min/session; cathode: contralateral cheek). At follow-up, no tDCS.fNIRS recorded on bilateral PFC (F3, F4) during 2-back task performance.	Trained tasks = visual recall task (5 items were presented for memorization, followed by a delay period. Participants were asked to recognize them in a 16-item array) and visuospatial recall tasks (3 items were presented for memorization, followed by a delay period. Participants were asked to recognize and localize them in a 12-item array). Near transfer task: visual 0- and 2-back task.	Pre-intervention and 1-month follow-up.	By collapsing trained, near transfer, far transfer tasks to obtain composite indexes, no group differences in the WM trained or near transfer tasks; 2 mA tDCS produced greater standard and ecologically valid far transfer gains than sham, whereas 1 mA showed a significant advantage only on in one ecologically valid task (OT-DORA). For the 2-back fNIRS measure, a greater percentage of R-PFC channels showing decreased activation from pre–intervention to follow-up was positively associated with 2-back performance, irrespective of tDCS group.
	Teixeira-Santos et al. (2022) [56]	54 OA (M = 68.2, SD = 5.92).	5-session WM intervention combining WM training with online anodal tDCS; between-subjects design: active tDCS + WM training, sham + WM training, sham + visuoperceptual task (control). Active stimulation: 2 mA to L-dlPFC (F3) × 20 min/session; cathode: contralateral supraorbital area.	Trained task = adaptive dual n-back task, with simultaneously visuospatial and auditory-verbal stimuli; the n-back level continued adaptively from the previous training session.	Pre-, during, and post-intervention, 15-day follow-up.	At baseline, the sham + WM training group performed at a higher level on the trained dual n-back task than the active tDCS + WM training group. Both active tDCS + WM training and sham + WM training groups improved on the trained dual n-back task, with no significant between-group difference. Far-transfer effects were observed for reasoning (RAPM), particularly in the active tDCS + WM training group, but no consistent tDCS advantage emerged on near-transfer Digit Span measures. Gains in backward Digit Span predicted gains in reasoning within the active tDCS + WM training group.

Note. CC = contralateral cheek; dlPFC = dorsolateral prefrontal cortex; EEG = electroencephalography; fMRI = functional magnetic resonance imaging; fNIRS = functional near-infrared spectroscopy; HD = high-definition; IPL = inferior parietal lobule; L = left; M = mean of age; MFG = middle frontal gyrus; OA = healthy older adults; offline = stimulation delivered before/after task execution; online = stimulation delivered during task execution; oxy-Hb = oxygenated hemoglobin; PAC = phase-amplitude coupling; PFC = prefrontal cortex; PPC = posterior parietal cortex; R = right; rMT = resting motor threshold; RT = reaction time; SD = standard deviation of age; tACS = transcranial alternating current stimulation; TC = temporal cortex; tDCS = transcranial direct current stimulation; TMS = transcranial magnetic stimulation; tRNS = transcranial random noise stimulation; VR = virtual reality; vWM = visual working memory; WM = working memory; YA = younger adults.

## Data Availability

No new data were created or analyzed in this study. Data sharing is not applicable to this article.
